# Acute Recovery of Oral Word Production Following Stroke: Patterns of Performance as Predictors of Recovery

**DOI:** 10.3233/BEN-2009-0245

**Published:** 2009-12-07

**Authors:** Lauren Cloutman, Melissa Newhart, Cameron Davis, Jennifer Heidler-Gary, Argye E. Hillis

**Affiliations:** ^1^Departments of NeurologyJohns Hopkins University School of MedicineBaltimoreMDUSA; ^2^Physical Medicine and RehabilitationJohns Hopkins University School of MedicineBaltimoreMDUSA; ^3^Department of Cognitive ScienceJohns Hopkins UniversityJohns Hopkins University School of MedicineBaltimoreMDUSA

## Abstract

*Background:* Impairments in oral word production are common at the onset of stroke. The identification of factors that predict early recovery has important implications for identifying those at greater risk of continued impaired functioning, and the management of the patient's care following discharge.

*Aims:* To identify patterns of performance that are predictors of acute recovery of oral word production abilities following stroke; to identify any association between early and more chronic recovery.

*Method and procedures:*Acute stroke patients were administered oral word production tasks within 1–2 days of hospital admission, with repeat testing by 7 days; a subset of patients had repeat testing between three weeks to one year later. Performance was examined for error rate and type to identify potential predictors of early recovery.

*Outcome and results:* The proportion of circumlocution and no response errors at initial testing were associated with the magnitude of recovery of language functioning within the first week following stroke. Patient characteristics of age and gender were found to have no influence on the degree of early recovery observed. None of the examined factors predicted late recovery. The degree of early recovery was not associated with the degree of later recovery.

*Conclusions:* The current study identified patterns of task performance that increase our understanding of how oral word production recovers following acute stroke. The finding that the degree of early recovery does not predict the degree of later recovery is consistent with the hypothesis that early and late recovery are due to different mechanisms (restored blood flow in acute stroke, and reorganization in later recovery).

### 1. Introduction

Impairments in oral word production are commonly observed in the acute presentation of stroke, with deficits found across a range of language tasks including oral naming, oral reading, and repetition (see Ferro [5], for a review). Recovery of such impairments, either spontaneously or following therapy, is frequently observed, with the greatest degree of recovery found within the first few weeks and months following stroke [4,6,16,17,19–21]. However, although some recovery of word production appears to be common, there is a great deal of variability in the degree of recovery observed, with some patients showing no improvement in functioning or even demonstrating a decline in performance [13,17]. The rate of early recovery might predict the rate of later recovery. While this hypothesis is intuitively plausible, it appears not to have been tested empirically.

A number of previous studies have examined the recovery of language (and other cognitive) functioning

ISSN 0953-4180/09/$17.00 © 2009 – IOS Press and the authors. All rights reserved following stroke in an attempt to understand who is likely to recover and why. The majority of these previous studies have focused on the examination of neural correlates associated with improved functioning([1,7, 14,18,23], for a review). Other studies have attempted to find correlates of recovery by examining specific patient characteristics, such as age, gender, education, and lesion location/size, with inconsistent findings (e.g., age: Nys et al. [19], compared with Lazer et al. [17]; gender: Nys et al. [20], compared with Hochstenbach et al. [13]).

Few studies have examined cognitive factors associated with the degree of recovery of language (or other cognitive) functioning [17,19–21]. Those that have attempted to do so have focused on overall initial task performance or severity of impairment as a potential predictor of the degree of language recovery. These studies have found that, while baseline cognitive performance is a significant predictor of future recovery, it only accounts for a small portion of the variance; and even patients with a similar degree of initial impairment can vary widely in their degree of future recovery [17].

The current study aimed to further our understanding of the recovery of oral word production by examining patterns of task performance that are potentially associated with recovery in the acute phase (i.e., within the first seven days following stroke onset), and whether or not the rate or characteristics of early recovery predict later recovery. To do so, we examined the accuracy and error patterns on verbal naming, oral reading, and repetition tasks, produced by individuals within the first 48 hours of stroke onset, and how these patterns of performance were associated with performance on these same tasks on repeat testing in the first week. In a subset of patients, we examined the relationship between early recovery and recovery between three months to one year later.

### 2. Method

#### *2.1. Participants*

A series of 99 right-handed patients with symptoms of left hemisphere acute ischemic stroke, were initially enrolled upon meeting the following inclusion criteria: premorbid proficiency in English, no known hearing loss or uncorrected visual impairment; no history of dementia, previous symptomatic stroke, or other neurological disease; and no hemorrhage on initial scans. Time 1 testing was attempted to be completed within 24 hours of stroke onset; however, some patients were included who were tested between 24 and 48 hours of stroke onset (usually because they were admitted close to or after 24 hours after initial symptoms). Following enrollment, 29 patients were excluded from the current study due to the absence of infarct on MRI or CT scans, failure to produce any intelligible verbal response on any of the tasks examined at Time 1 testing, or the lack of repeat testing on the language tasks of specific interest (oral naming, oral reading, repetition). The remaining group of 70 patients consisted of 41 men and 29 women. The mean age was 62.4 $\pm$ 15.1 standard deviation (SD) years; the mean education level was 11.5 $\pm$ SD 3.1 years. Of the 70 patients, 29 were found to have either bilateral infarcts, and/or previous (asymptomatic) stroke, and/or repeat testing at the subacute ($>$7 days) stage. While an initial regression analysis included these patients to examine any influence of these factors, the main analyses were conducted on the subgroup of 41 patients who had purely left hemisphere infarcts, with no history of prior stroke by MRI, and whose repeat testing was within the time period of interest (the 'Acute Left First Stroke' group; 24 men, 17 women; mean age 63.4 $\pm$ 14.0 SD years; mean education level 11.4 $\pm$ SD 3.3 years). Finally, a group of 8 patients completed Time 3 testing 3-12 months later (7 men, 1 woman; mean age 66.3 $\pm$ 8.97 SD years).

#### *2.2. Language tasks*

##### *2.2.1. Oral naming tasks*

Patients completed a noun naming task in which they were presented with pictures of objects for untimed oral naming, with line drawings taken from Snodgrass and Vanderwart [24]. Some patients named 30 pictured objects, but the majority named only a smaller set of 17. The stimulus sets were matched for word length, frequency, and semantic categories. A subset of patients also completed a verb naming task, in which patients orally named 30 pictured actions. These verbs were matched in length and frequency to the 30 item noun naming task. Finally, patients completed a tactile naming task, in which they were required to name 17 objects identified by touch alone.

##### *2.2.2. Reading task*

Patients were presented with a 58 item oral reading task, involving both word and nonword reading. [1] For

[1] Patients required at least a 10th grade education level to be administered the reading task.

the majority of patients, the task involved reading 34 words and 24 nonwords; however for a small subset of patients, the task involved reading 36/22 or 35/23 words/nonwords, due to an error in the stimuli. Words ranged in length from three to seven letters (mean = 5 ± SD 1.3 letters), while nonwords tended to be slightly shorter, ranging from three to five letters (mean = 4.1± SD 0.7 letters).

#### *2.2.3. Repetition task*

Patients were presented with a 58 item repetition task, involving the repetition of auditorally-presented words and nonwords. As with the reading task, the task involved reading 34 words and 24 nonwords. (However, for a small subset of patients, the task involved reading 36/22 or 35/23 words/nonwords, due to an error in the stimuli). Words ranged in length from three to nine phonemes (mean = 4.2 ± SD 1.5 phonemes), while nonwords tended to be slightly shorter, either three or four phonemes in length (mean = 3.3 ± SD 0.5 phonemes).

Time 2 tests were identical to Time 1 tests, but with separate stimuli that were matched in word length, word frequency, and word class to the Time 1 stimuli. Time 3 tests were also identical to Time 1 tests, but with a third set of stimuli that were matched in word length, word frequency, and word class to the Time 1 and Time 2 stimuli.

### *2.3. Data analysis*

On all tasks, the initial response was scored. Performance was coded for word/nonword error rate and type. Error categories for noun naming, and word reading/repetition included: 1) semantic, which included coordinates (e.g. "cat" for the target word 'dog'), associates (e.g., bone for dog), superordinates (e.g., animal for dog), and subordinates (e.g., Labrador for dog); 2) circumlocutions (e.g., they bark a lot); 3) phonemic (nonwords related to the target by initial or final phoneme, e.g., dop for dog); 4) formal/visual (words related to the target by initial or final letter, e.g., dot for dog); 5) neologisms (nonwords phonologically unrelated to the target, e.g., mip for dog); 6) morphological (e.g., dogs for dog); 7) mixed, which bore both a semantic and phonological/visual relationship to the target (e.g., frog for dog); 8) unrelated (e.g., table for dog); 9) no responses; and 10) other errors, which could not be categorized (which predominantly consisted of word fragments). The error categories for nonword reading/repetition were the same as those for word reading, with the exclusion of semantic, circumlocutions, mixed, and morphological errors (which were not possible for nonwords).

Stepwise multiple linear regression analyses were conducted to identify any patient/performance variables which were predictive of either: a) the magnitude of change in total percent correct between Time 1 testing and Time 2 testing ('Early Change Score'); and b) the accuracy of performance at Time 2 ('Time 2 Accuracy Score'). Variables included in the analysis included: age, gender, stroke hemisphere (left or bilateral), history of prior strokes, time between testing (in days), Time 1 performance accuracy, and the error rates at Time 1 for the individual error categories described above.[2]

We evaluated the relationship between early and later recovery by graphing the rate of change in percent correct from Time 1, Time 2, and Time 3 for each patient to evaluate whether the slope of change in the acute stage was related to the slope of change during later recovery or the final accuracy. We also conducted a regression analysis, with a) the magnitude of change in total percent correct between Time 1 testing and Time 3 testing ('Late Change Score'); and b) the accuracy of performance at Time 3 ('Time 3 Accuracy Score') as the dependent variables, and time between Time 1 and Time 3, Early Change Score (at Time 2), and Time 1 and Time 2 accuracy scores as the independent variables.

## 3. Results

### *3.1. Whole group analysis*

Of the 70 patients, 47 (67.1%) showed some degree of improvement in overall performance between the Time 1 and Time 2 testing sessions. Looking first at the Early Change Score analysis, only Time 1 performance accuracy [$\beta = -0.69$, $t(67) = -6.31$, $p < 0.001$], and the Time 1 neologism error category [$\beta = -0.44$, $t(67) = -4.06$, $p < 0.001$], were significantly associated with the magnitude of change in overall performance between the two testing sessions. That is, higher accuracy and higher proportion of neologisms predicted a lower slope of early recovery. There was a negative relationship between both these variables and the magnitude of the Early Change Score. These two

[2]Education was not included in the regression analysis due to missing data for some patients.

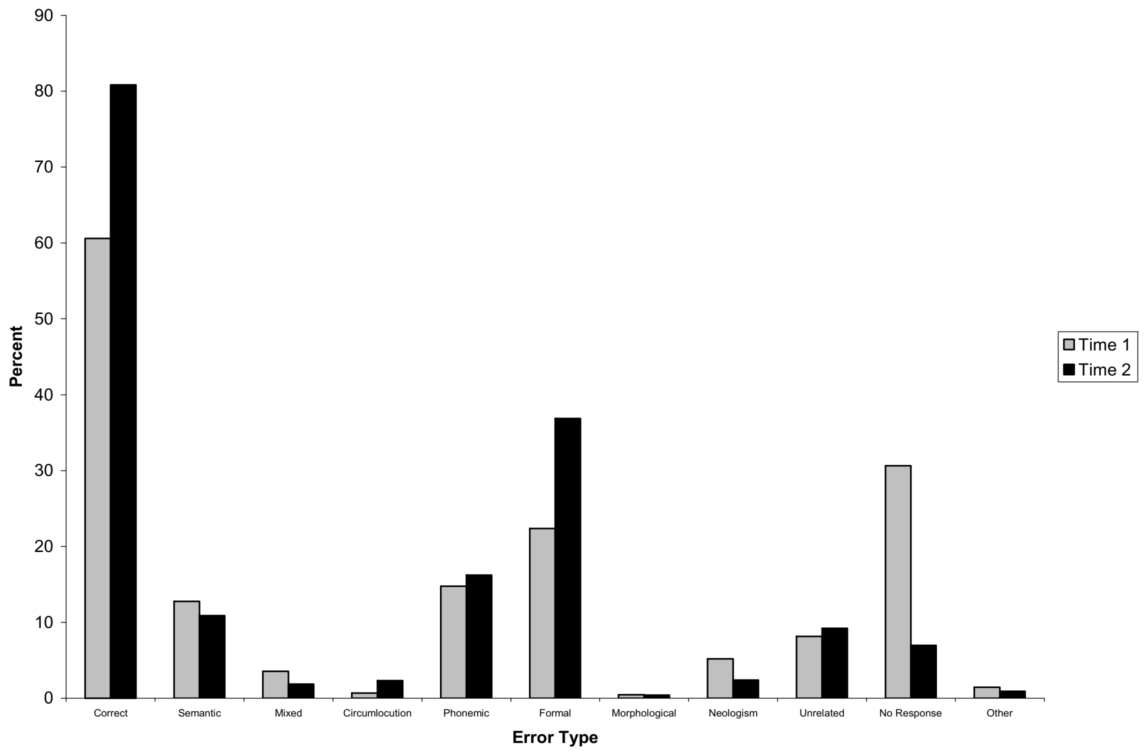

Fig. 1. Accuracy (as percentage of total responses) and error rates (as percentage of total errors) for the Acute Left First Stroke group who demonstrated improvement.

predictor variables were able to account for approximately one-third of the variance in the Early Change Score [Adjusted $R^2$ = 0.36, $F$(2, 67) = 20.69, $p <$ 0.001].

Moving on to the Time 2 Accuracy Score analysis, as with Early Change Score, only Time 1 performance accuracy [$\beta$ = 0.54, $t$(67) = 6.06, $p <$ 0.001], and the Time 1 neologism error category [$\beta = -$ 0.35, $t$(67) $= -$ 3.93, $p <$ 0.001], were significantly associated with the performance accuracy rate at Time 2. Not surprisingly, Time 1 performance accuracy was found to have a positive relationship with Time 2 performance accuracy, while Time 1 neologisms had a negative relationship. That is, higher accuracy at Time 1 predicted higher accuracy at Time 2, while a higher proportion of neologisms at Time 1 predicted a lower accuracy at Time 2. These two predictor variables were able to account for over half of the variance in the Time 2 Accuracy Score [Adjusted $R^2$ = 0.58, $F$(2, 67) = 47.59, $p <$ 0.001].

#### 3.2. *Acute left first stroke group analysis*

Of the 41 patients, 25 (61%) showed some degree of improvement in overall performance between the Time 1 and Time 2 testing sessions. Figures 1 and 2 display the accuracy and error rates for the group of patients who improved in performance across the two testing sessions (Fig. 1) and for the group who did not improve (Fig. 2). Examination of Fig. 1 indicates that those who improved showed a reduction in the proportion of a number of error types across the two testing sessions, which was significant for neologisms [$t$(24) = 2.12, $p <$ 0.05], and no response errors [$t$(24) = 4.51, $p <$ 0.001], but actually showed an increase in the proportion of formal errors between the two testing sessions [$t$(24) $= -$ 2.6, $p <$ 0.05]. In contrast, the non-improvers (Fig. 2) were more variable in the changes in error types across testing sessions, with no significant differences in the proportions of any error types between sessions.

There were no differences between the improvers and non-improvers in the initial Time 1 accuracy rates or proportions of any error types produced at Time 1. However, significant differences were found between these two groups for the degree of change between Time 1 and Time 2 in the proportion of no response [$t$(39) = $-$3.59, $p <$ 0.01] errors. Looking at Figs 1 and 2, the difference in the change in the proportion

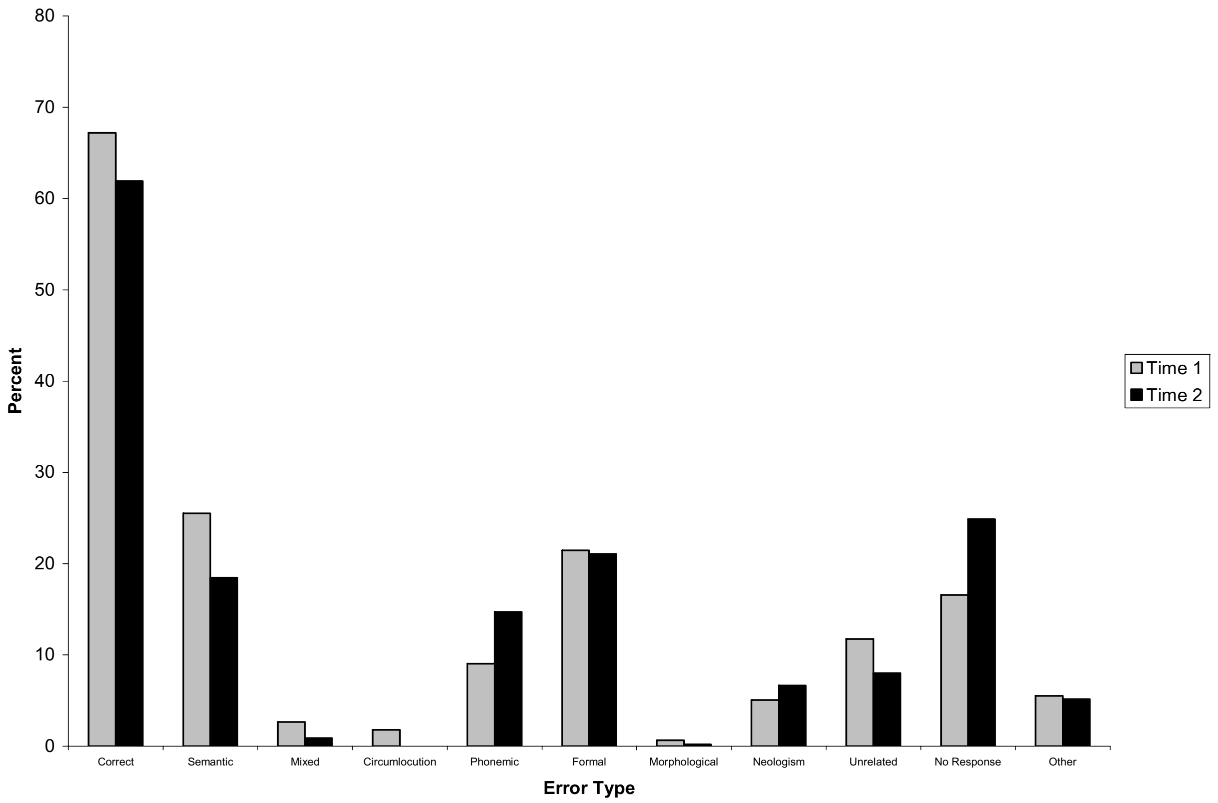

Fig. 2. Accuracy (as percentage of total responses) and error rates (as percentage of total errors) for the Acute Left First Stroke group who did not improve.

of no response errors seems to result from the fact that those patients who improved showed a reduction in these error types across testing sessions, while those who did not improve produced an increased proportion of no response errors at Time 2.

Stepwise multiple linear regression analyses were conducted to identify any patient/performance variables that predicted either the Early Change Score or the Time 2 Accuracy Score. Variables included in the analysis included: age, gender, time between testing (in days), Time 1 performance accuracy, and the error rates at Time 1 for the individual error categories described previously. Looking first at the early Change Score analysis, only Time 1 circumlocution [$\beta = 0.31$, $t(38) = 2.26$, $p < 0.05$], and no response [$\beta = 0.44$, $t(38) = 3.25$, $p < 0.01$] error categories were significantly associated with the magnitude of change in overall performance between the two testing sessions. There was a positive relationship between both these variables and the magnitude of the Early Change Score. These two predictor variables were able to account for approximately one-third of the variance in the Early Change Score [Adjusted $R^2 = 0.34$, $F(2, 38) = 11.1$, $p < 0.001$].

Moving on to the Time 2 Accuracy Score analysis, only Time 1 performance accuracy [$\beta = 0.58$, $t(37) = 4.94$, $p < 0.001$], Time 1 neologism [$\beta = -0.37$, $t(37) = -3.25$, $p < 0.01$], and Time 1 circumlocution [$\beta = 0.2$, $t(37) = -2.05$, $p < 0.05$] error categories were significantly associated with the performance accuracy rate at Time 2. As we found for the total group of patients (including those with bilateral or previous stroke), Time 1 performance accuracy by those with First Left Stroke was found to have a positive relationship with Time 2 performance accuracy, while Time 1 neologisms had a negative relationship. These two predictor variables were able to account for approximately two-thirds of the variance in the Time 2 Accuracy Score [Adjusted $R^2 = 0.66$, $F(2, 37) = 27.37$, $p < 0.001$].

Finally, a generalized linear analysis which corrected for repeated measures was conducted to identify any differences in the degree of change in performance across the different tasks examined. This analysis indicated that there was no difference in the magnitude of improvement across the three oral word production

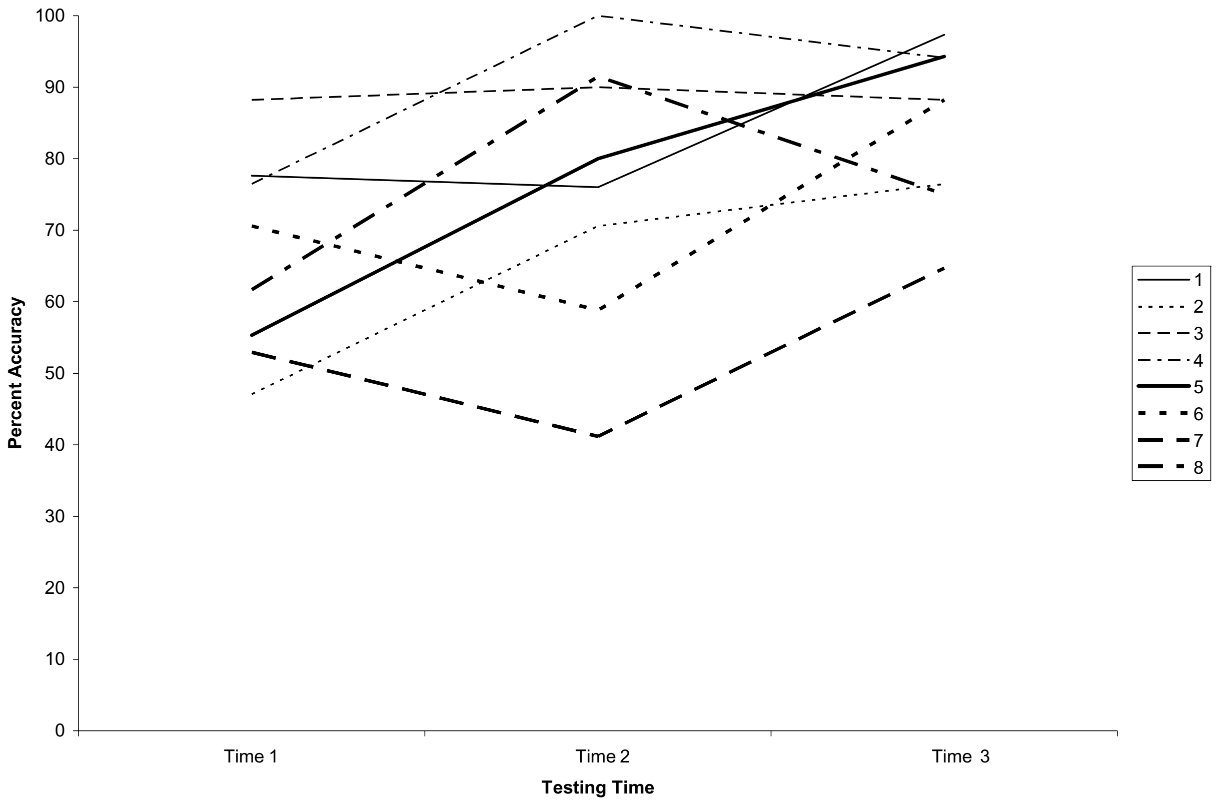

Fig. 3. Accuracy rates across the three testing periods for each of the eight patients examined.

tasks examined (oral naming, oral reading, or repetition).

#### 3.3. *Late recovery analysis*

A subset of 8 patients returned for Time 3 testing between 3 weeks to one year after stroke. Accuracy scores at the three time points are shown in Fig. 3 for each of these patients. A linear regression analysis indicated that there were no predictors of Late Change Score, or Time 3 Accuracy.

#### 3.4. *General discussion*

The current study examined accuracy rates and error patterns on oral word production tasks in an attempt to identify possible correlates and predictors of the recovery of verbal language functioning in the early stages following stroke. The majority of patients in the current study showed some degree of recovery in overall word production performance, although there was wide variability in the degree of recovery observed. Previous studies have noted that the greatest degree of recovery occurred within the first few weeks or months post-stroke [4,16,17]. The current study extends these findings to demonstrate recovery within the first week post-stroke, and (in a small subset of patients) how early recovery is related to later recovery.

Patient characteristics (which included age, gender, history of prior stroke, and infarct hemisphere), and task type were found to have no influence on the recovery of oral word production, demonstrating no effect on the magnitude of change in performance between the first two testing sessions. In contrast, there were some specific error patterns produced at Time 1 that were found to be associated with the degree of early recovery. Specifically, the proportion of circumlocution and no response errors were found to be associated with the magnitude of recovery. In addition, initial accuracy rates and the proportion of neologism and circumlocution errors at Time 1 testing were both found to be associated with the accuracy of performance at Time 2.

None of the patient characteristics in the current study were found to have an influence on the magnitude of change in performance between the testing sessions. Previous studies have demonstrated inconsistent findings in relation to their influence on recovery. For example, some studies have found that patients who were older demonstrated less cognitive recovery than those who were younger (e.g. [16,19,20]), while other studies have failed to find an effect of age on the degree of recovery observed (e.g. [17]). Variability in the types of task examined, the specific language domains examined (i.e., production versus comprehension), and the time frame of recovery across studies may account for some of the inconsistencies in findings. It appears from the current study that in the acute stage of recovery at least, patient characteristics such as age and gender do not influence the degree of recovery of oral word production in tasks such as naming, repetition, and oral reading. There were too few patients tested in later recovery to draw conclusions regarding the effect of these variables on later recovery. Older patients live for a shorter time after stroke and therefore have less time to recover.

In contrast to the patient characteristics, an examination of error patterns did reveal some associations between the types of errors produced and the degree of recovery observed within the first week following stroke. The proportion of circumlocutions and no response errors produced at Time 1 testing both predicted the magnitude of early recovery observed, and were both found to have a positive relationship with recovery (Early Change Score); that is, greater proportions of circumlocution and no response errors at Time 1 were associated with greater magnitude of change. It is interesting to note that initial severity of impairment was not associated with the magnitude of change in First Left Stroke. This is consistent with previous studies which have found that patients are as likely to recover from a severe deficit as a less severe one (e.g. [17]). Thus, the association between the degree of recovery and the proportion of no response and circumlocution errors is not the result of these error types being associated with a more severe impairment overall, and therefore a greater potential for early recovery.

One benefit of examining error patterns associated with recovery is that not only can we identify possible predictors of recovery, but we can also explore possible underlying mechanisms by which this recovery may be occurring. What, then, can we make of the association between degree of recovery and the proportion of circumlocution and no response errors produced? Researchers have noted similarities between circumlocution and no response errors in that both error types are associated with a failure to access or select any phonological information associated with the target, although with circumlocutions, semantic information about the concept to be produced is available [2,15].

One possible account of early recovery in these patients is that it may reflect an improvement in accessing and/or selecting the phonological information associated with the target concept, resulting in improved performance. Thus, those patients who demonstrated the greatest impairment in accessing any phonological information, by way of the production of high rates of no response and circumlocution errors, had the greatest potential for improving. Interestingly, although not found to be a predictor of recovery, the proportion of formal errors between testing sessions actually increased for the group of patients who improved overall, while this was not found for those who failed to improve. It is possible that as patients recover, they are able to access greater phonological information associated with the target, resulting in improved overall performance and a shift in error type to real word errors which are phonologically associated with the target. Of course, it should be noted that this proposal is highly tentative, and further examination with a greater number of patients is required before any firmer conclusions can be made. Rather, the current account is put forward as an example of the possible advantages of examining not just accuracy but error patterns in attempting to understand and predict patterns of recovery of language functioning following stroke.

Early recovery in these patients did not reflect results of language therapy, as none of the patients had language therapy in the first week. Our previous studies indicate that acute recovery (in the first week) results primarily via tissue reperfusion, through spontaneous or elicited restoration of blood flow. Blood flow can be restored through a variety of interventions, such as thrombolysis, stenting, endarterectomy, or pharmacologically induced blood pressure elevation (see [8, 11,12]). The current study suggests that blood flow changes in early stroke are most likely to occur in areas essential for accessing phonological (or modality independent) word forms, resulting in increased correct responses and formal errors, and a reduction in no response errors and circumlocutions. The fact that early recovery likely resulted from restored blood flow, while later recovery likely reflected reorganization of structure/function relationships (see [9]) likely explains why the rate and characteristics of early recovery did not predict the magnitude of later recovery.

Despite being significant predictors or early recovery, error patterns at Time 1 testing were only able to account for approximately one-third of the variance in recovery observed in the current study. In contrast, the current study demonstrated a much greater ability to predict the level of performance on these tasks at Time 2, with initial accuracy and the proportion of neologism and circumlocution errors produced at Time 1 able to account for approximately two-thirds of the variance in Time 2 performance. Although the positive relationship between the accuracy at Time 1 and the accuracy at Time 2 is not surprising, the current study also demonstrated that there was a negative relationship between the proportion of neologisms produced at Time 1 and accuracy at Time 2. Neologisms are associated with greater disruption to the language production system than other errors [3]. Thus, although severity of impairment at Time 1 is not associated with the overall degree of improvement (magnitude of change), it is associated with the ability to produce the target at Time 2. Patients who do not have a high accuracy rate and produce a high proportion of neologistic errors at Time 1 are not likely to perform well at Time 2 testing.

Only a small number of patients completed Time 3 testing, making it difficult to draw conclusions regarding any relationship between initial performance and later recovery. However, it is interesting to note that all of the chronic patients examined demonstrated improvement at Time 3 compared to initial testing, regardless of whether or not they showed improved performance at Time 2. Thus, the degree of initial improvement may not be an indication of the degree of long-term improvement at more chronic stages of recovery, and patients can deteriorate after showing initial improvement, and vice versa.

#### 3.5. *Conclusion*

While the exploration of neural and clinical predictors of cognitive recovery following stroke has increased our knowledge regarding potential correlates of recovery, cognitive predictors of early recovery and their relationship to later recovery have previously been unexplored. The current study identified patterns of task performance that increase our understanding of how oral word production recovers following acute stroke. The result that the degree of early recovery does not predict the degree of later recovery is consistent with the hypothesis that early and late recovery are brought about by very different mechanisms. Our previous work and that of others (see Hillis [9] for review) indicates that the primary mechanism of acute recovery of language is restored blood flow to critical areas of the brain, and that the primary mechanism of late-stage recovery is reorganization of structure/function relationships. Our study suggests that the latter can occur independently of whether or not there was restored blood flow in the acute stage.

### Acknowledgements

The research reported in this paper was supported by NIH (NIDCD), through RO1 DC 05375. We gratefully acknowledge this support and the participation of the patients.
